# FDG-PET Profiles of Extratemporal Metabolism as a Predictor of Surgical Failure in Temporal Lobe Epilepsy

**DOI:** 10.3389/fmed.2020.605002

**Published:** 2020-12-14

**Authors:** Yongxiang Tang, Guang Liao, Jian Li, Tingting Long, Yulai Li, Li Feng, Dengming Chen, Beisha Tang, Shuo Hu

**Affiliations:** ^1^Department of Nuclear Medicine, Xiangya Hospital, Central South University, Changsha, China; ^2^Department of Neurology, Xiangya Hospital, Central South University, Changsha, China; ^3^National Clinical Research Center for Geriatric Diseases, Xiangya Hospital, Central South University, Changsha, China; ^4^Key Laboratory of Biological Nanotechnology of National Health Commission, Xiangya Hospital, Central South University, Changsha, China

**Keywords:** PET in epilepsy, prognosis, epilepsy, image processing, PET

## Abstract

**Objective:** Metabolic abnormality in the extratemporal area on fluorine-18-fluorodeoxyglucose positron emission tomography (FDG-PET) is not an uncommon finding in drug-resistant temporal lobe epilepsy (TLE), however the correlation between extratemporal metabolic abnormalities and surgical long-term prognosis has not been fully elucidated. We aim to investigate FDG-PET extratemporal metabolic profiles predictive of failure in surgery for TLE patients.

**Methods:** Eighty-two patients with unilateral TLE (48 female, 34 male; 25.6 ± 10.6 years old; 37 left TLE, 45 right TLE) and 30 healthy age-matched controls were enrolled. Patients were classified either as experiencing seizure-recurrence (SZR, Engel class II through IV) or seizure-free (SZF, Engel class I) at least 1 year after surgery. Regional cerebral metabolism was evaluated by FDG-PET with statistical parametric mapping (SPM12). Abnormal metabolic profiles and patterns on FDG-PET in SZR group were evaluated and compared with those of healthy control and SZF subjects on SPM12. Volume and intensity as well as special brain areas of abnormal metabolism in temporal and extratemporal regions were quantified and visualized.

**Results:** With a median follow-up of 1.5 years, 60% of patients achieved Engel class I (SZF). SZR was associated with left TLE and widespread hypometabolism in FDG-PET visual assessment (*both p* < 0.05). All patients had hypometabolism in the ipsilateral temporal lobe but SZR was not correlated with volume or intensity of temporal hypometabolism (median, 1,456 vs. 1,040 mm^3^; *p* > 0.05). SZR was correlated with extratemporal metabolic abnormalities that differed according to lateralization: in right TLE, SZR exhibited larger volume in extratemporal areas compared to SZF (median, 11,060 vs. 2,112 mm^3^; *p* < 0.05). Surgical failure was characterized by Cingulum_Ant_R/L, Frontal_Inf_Orb_R abnormal metabolism in extratemporal regions. In left TLE, SZR presented a larger involvement of extratemporal areas similar to right TLE but with no significant (median, 5,873 vs. 3,464 mm^3^; *p* > 0.05), Cingulum_Ant_ R/L, Parietal_Inf_L, Postcentral_L, and Precuneus_R involved metabolic abnormalities were correlated with SZR.

**Conclusions:** Extratemporal metabolic profiles detected by FDG-PET may indicate a prominent cause of TLE surgery failure and should be considered in predictive models for epilepsy surgery. Seizure control after surgery might be improved by investigating extratemporal areas as candidates for resection or neuromodulation.

## Introduction

The goal of epilepsy surgery is to render the patient seizure-free. Surgical treatment of drug-resistant temporal lobe epilepsy (TLE) has proven superior to medical management of the disease (1). However, the proportion of seizure-free following TLE surgery remains suboptimal (2, 3). In ~50% of cases in which surgery fails to achieve seizure freedom, patients who continue to experience seizures after surgery are directly associated with an even lower quality of life (4). It has proven difficult to identify from existing literature independent biomarker highly predictive of TLE surgery failure. Therefore, understanding the prominent reasons for surgical failure and identifying effective indicators to facilitate early evaluation remain of paramount importance in the context of epilepsy care (3, 5–7).

Evidence from neuroimaging and electrophysiology studies has consistently shown that epilepsy is a disease affecting neural networks, with abnormalities occurring well-beyond the locus of ictogenesis (8). While recurrent seizures following surgery suggested that some epileptogenic tissue distinct from the primary temporal lobe epileptogenic zone has not been resected, and those areas might reflect dual pathology beyond seizure onset zone, but usually negative in magnetic resonance imaging (MRI) or impossibility of whole brain electrocorticogram (ECoG) coverage (5, 7), thus posing a difficult diagnostic challenge. Emerging evidence suggests that temporal lobe hypometabolism on fluorine-18-fluorodeoxyglucose positron emission tomography (FDG-PET) can provide relevant information on the epileptogenic zone extent and surgical outcome (9–11). The highest clinical benefit of FDG-PET can be achieved in patients with MRI-negative TLE (12). However, metabolic abnormality in the extratemporal cortex on FDG-PET is not an uncommon finding and seems to be associated with surgical outcome in TLE (13, 14), but the correlation between extratemporal metabolic abnormalities and surgical long-term prognosis has not been fully elucidated. Moreover, it is not clear which profiles of temporal or extratemporal metabolic abnormalities are more important for TLE surgical failure.

In this study, we analyzed patients with unilateral TLE who had undergone identical surgical resections. Long-term seizure outcomes were analyzed to determine the potential usefulness of FDG-PET temporal and extratemporal metabolic intensity, volume and/or specific brain areas for predicting postoperative seizure recurrence in TLE patients. Imaging processing was performed with statistical parametric mapping (SPM 12).

## Materials and Methods

### Patients and Healthy Controls

We retrospectively reviewed 82 drug-resistant unilateral TLE patients (45 right TLE [RTLE], 37 left TLE [LTLE]) who had received preoperative FDG-PET between April 2014 and April 2018. Diagnosis of drug-resistant unilateral TLE was based upon comprehensive clinical assessment and criteria of the International League Against Epilepsy (ILAE) (15). Each patient was surgically treated by identical anteromedial temporal resection (AMTR) without extratemporal resections as described by Spencer et al. (16). Pathology was assessed from postoperative pathology reports. The determination of postsurgical outcome was based on in-person interviews and patient assessment during clinic follow-up. Patients without 1-year follow-up were excluded from analysis.

Thirty healthy age-matched volunteers were recruited as normal controls. None had a history of head injury or a major neurological, physical, or psychiatric disorder, including drug and alcohol misuse.

### Ethical Approval and Patient Consent

Study protocols was approved by the Ethical Commission of Medical Research Involving Human Subjects at Region of Xiangya Hospital, Central South University, China [IBR{C}NO. (201412455)]. Written informed consent was provided by all participants (patients and controls) in accordance with the Helsinki Declaration.

### Clinical Data

In addition to undergoing FDG-PET, patients completed a pre-surgical assessment consisting of a detailed clinical history and examination, video electroencephalogram (EEG) monitoring, brain MRI, neuropsychiatric testing and intracranial EEG monitoring when indicated. FDG-PET classification was defined as previously described (17) and classified into two subtypes according to the visual assessment: focal or widespread hypometabolism.

Outcome assessments were performed 3 and 12 months after surgery and at yearly intervals thereafter. All patients were interviewed in detail for seizure recurrence, if any, and date of recurrence. Surgical outcomes were classified based on the Engel Surgical Outcome scale (18, 19) as either seizure-free (SZF; Engel class I) or seizure-recurrence (SZR; Engel class II through IV).

### FDG-PET Image Acquisition and Processing

FDG-PET was acquired using a Discovery Elite PET/CT scanner (GE Healthcare) prior to surgical resection. FDG was injected intravenously at a mean dose of 148 MBq. Images were acquired in three dimensions over a 60 min time period, following scanning protocol described by Tang et al. (11). Image processing was performed using the SPM12 (Wellcome Department of Cognitive Neurology, London, UK). Individual FDG-PET image volumes were spatially normalized into standard stereotactic Montreal Neurological Institute (MNI) space with voxel sizes of 2 × 2 × 2. An 8-mm full-width-half-maximum Gaussian kernel was used to improve between-participant spatial alignment and smooth data for statistical analysis. The right and left hemispheres were analyzed separately to detect lateralization effects on surgical outcomes (20). Image intensity between participants was normalized to prevent interparticipant variability in cerebral tracer uptake from masking regional changes. Increased or decreased metabolism was considered statistically significant when uncorrected *p* = 0.001 with cluster level above 20 contiguous voxels. After data preprocessing using SPM significant clusters were visualized, reported and anatomically labeled using the xjView (http://www.alivelearn.net/xjview), REST and BrainNet Viewer Toolkit (21, 22). Data include metabolic profile information about the clusters, including number of voxels (or volumes), anatomical term of Automated Anatomical Labeling (AAL) areas and peak intensity of each cluster.

### Statistical Analysis

All data were analyzed using SPSS software (IBM SPSS Statistics, Version 18.0). Numerical data are presented as mean ± *SD* or Median (IQR). Student *t*-tests or Mann-Witney tests and Pearson χ^2^ test were used for continuous and categorical variables in between-group comparisons, as appropriate. For FDG-PET image SPM analysis, the general linear model was used to carry out the appropriate voxel-by-voxel univariate statistical tests. Individual TLE SPM analysis was firstly performed using 30 healthy controls. The volume of metabolism changing in temporal, extratemporal areas and whole brain for each patient was then calculated as well as peak intensity for the ipsilateral temporal lobe. Abnormal metabolic volume in RTLE and LTLE was compared between two outcome groups using the Mann-Whitney test. Then, individual SZR SPM analysis was performed using SZF group, increased and decreased metabolism of extratemporal brain area were visualized and frequency was calculated. We then compared baseline glucose uptake values of each outcome group (SZR and SZF separately) and healthy controls in RTLE and LTLE using an analysis of covariance (ANCOVA) with group as the between-subject factor and age and sex as confounding covariates (23, 24). A two-sample *t*-test was used to compared the different groups. Other comparisons were performed between two outcome groups in both sides of TLE. Statistical significance was defined as *p* < 0.05.

## Results

### Clinical Data

Eighty-two refractory TLE patients (48 female, 59%) met the inclusion criteria of isolated AMTR with 1 or more years of follow-up. Median follow-up time was 1.5 years (IQR 1.2–2.3) with a maximum follow-up time of 5 years. Patient clinical characteristics are shown in Table 1. Briefly, 49 of 82 patients (60%) obtained an Engle class I outcome. SZF was more frequently obtained in RTLE (73%) than in LTLE (43%) and the difference was significant (*p* = 0.006). The main clinical variable differing significantly between SZF and SZR outcome groups were seen for focal or widespread hypometabolism on FDG-PET in RTLE. SZF group patients exhibited a narrower range of hypometabolism in FDG-PET analysis by visual assessment in RTLE (85.2% at focal hypometabolism vs. 55.6% widespread hypometabolism, *p* = 0.028). Regardless of whether there was hippocampal sclerosis(HS) in the postoperative histopathology or MRI, there was no difference in their surgical outcomes (*both p* > 0.05).

**Table 1:** Patient clinical characteristics and surgical outcomes.

	**LTLE patients (*****n*** **=** **37)**			**RTLE patients (*****n*** **=** **45)**			**All patients (*****n*** **=** **82)**		
	**SZF**	**SZR**	***p***	**SZF**	**SZR**	***P***	**SZF**	**SZR**	***p***
Gender (male/female), *n*	10/6	11/10	NS	20/13	7/5	NS	30/19	18/15	NS
Age (mean ±*SD*), y	24.8 ± 8.2	27.0 ± 12.1	NS	25.8 ± 11.2	23.8 ± 9.7	NS	25.5 ± 10.3	25.8 ± 11.3	NS
Age at onset (mean ±*SD*), y	10.7 ± 7.7	11.7 ± 8.3	NS	14.9 ± 11.1	9.7 ± 6.9	NS	13.5 ± 10.2	11.0 ± 7.8	NS
Duration of epilepsy (mean ±*SD*), y	14.4 ± 8.9	15.2 ± 8.6	NS	11.0 ± 7.7	14.2 ± 7.6	NS	12.1 ± 8.2	14.8 ± 8.1	NS
Surgicalside (L/R)	16 (43%)	21 (57%)	-	33 (73%)	12 (27%)	-	16/33(60%)	2112 (40%)	***p*** **<** **0.05**
**Histopathology**, ***n*** **(%)**			NS			NS			NS
HS	10 (27)	12 (32)		21 (47)	5 (11)		31 (38)	17 (21)	
Non-HS	6 (16)	9 (25)		12 (27)	7 (15)		18 (21)	16 (20)	
Handedness (L/R)	1/15	0/21	NS	1/32	0/12	NS	2/47	0/33	NS
**History**, ***n*** **(%)**			NS			NS			NS
Febrile seizures.	4 (11)	2 (5)		4 (9)	2 (5)		8 (10)	4 (5)	
Brain injury	1 (3)	4 (11)		1 (2)	0 (0)		2 (2)	4 (5)	
Without	10 (27)	15 (40)		24 (53)	9 (20)		34 (41)	24 (29)	
Encephalitis	1 (3)	0 (0)		4 (9)	1 (2)		5 (7)	1 (1)	
Psychiatric complication (with/without)	1/15	1/20	NS	0/33	1/11	NS	1/48	2/31	NS
Arua (with/without)	7/9	12/9	NS	18/15	6/6	NS	25/24	18/15	NS
Family history of epilepsy (with/without)	0/16	0/21	NS	0/33	1/11	NS	0/49	1/32	NS
Visual evaluation of PET (local/wide)	7/9	8/13	NS	23/10	4/8	**0.028**	30/19	12/21	**0.027**
Result of MRI (positive/negative)	13/3	14/7	NS	28/5	10/2	NS	41/8	24/9	NS
**Volume of metabolic change**									
Temporal areas, median (IQR)	1,040	1,056	NS	936	3,356	NS	1,040	1,456	NS
	(62–4,428)	(0–7,328)		(52–5,076)	(64–11,000)		(52–4,896)	(0–9580)	
Extratemporal areas, median (IQR)	3,464	5,872	NS	2,112	11,060	***p*** **<** **0.05**	2,112	8,424	***p*** **<** **0.05**
	(1,048–11,502)	(672–30,140)		(176–36,284)	(930–22,290)		(176–6,708)	(732–23,884)	

*p-value is derived from the univariable association analyses between each of the clinicopathologic variables and surgical outcome*.

*HS, hippocampal sclerosis; LTLE, left temporal lobe epilepsy; MRI, magnetic resonance imaging; NS, not significant; PET, positron emission tomography; RTLE, right temporal lobe epilepsy; SD, standard deviation; SZF, seizure-free (Engel class I); SZR, seizure recurrence (Engel class II through IV)*.

*Bold values indicates significant difference (p < 0.05)*.

### Volume of Metabolic Abnormalities in Temporal and Extratemporal Areas

**Figure 1:**
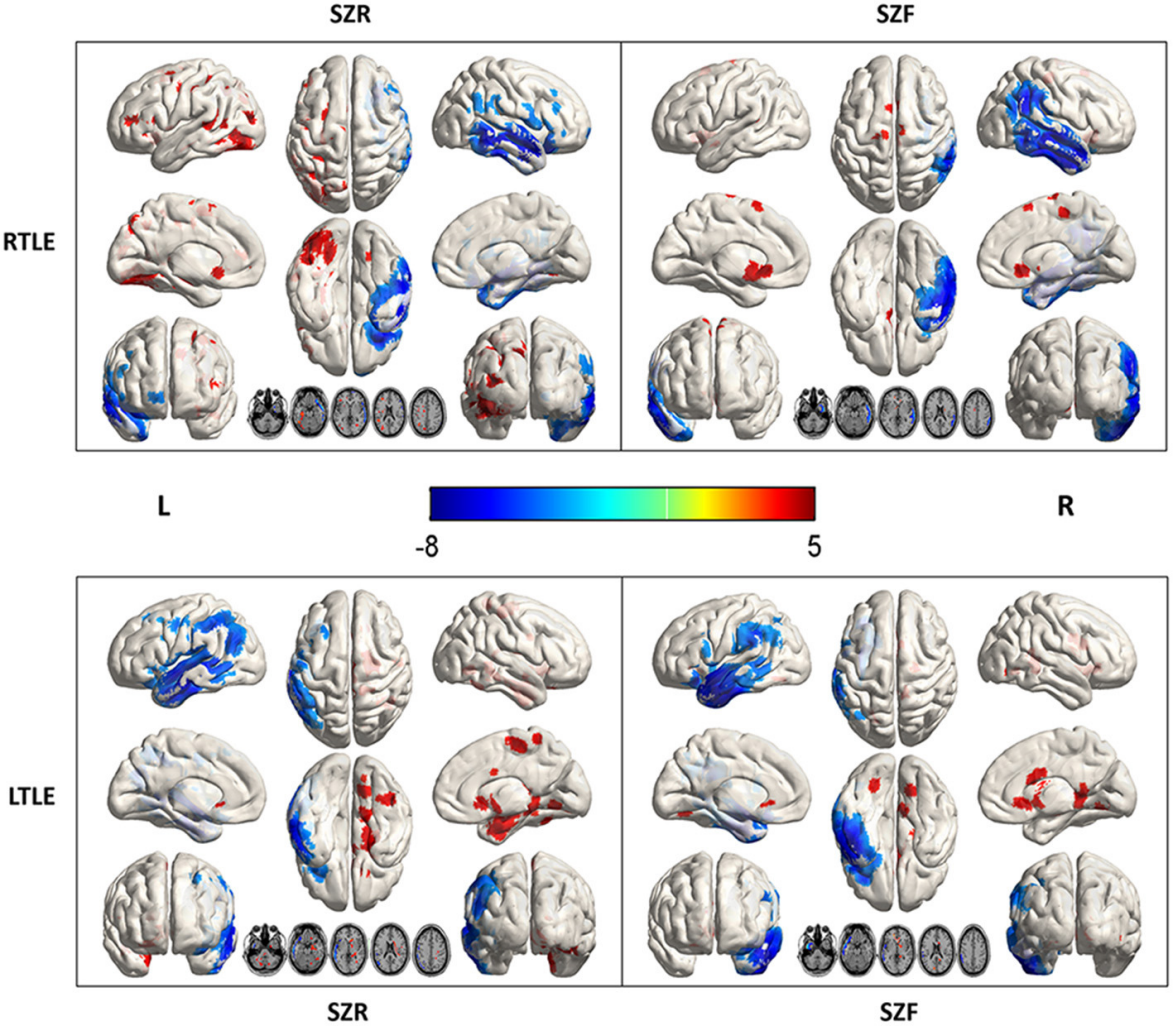
Metabolic data from 45 right (R) and 37 left (L) temporal lobe epilepsy (TLE) according to surgical outcome. Comparison with 30 healthy controls (SPM12, *p* < 0.001). In RTLE, hypometabolism (blue) was predominant in the temporal cortex ipsilateral to the epileptogenic region, with mild extratemporal involvement in the seizure-free group (SZF, Engel class I). In contrast, a larger volume involvement of nearby brain areas of foci and other perisylvian regions, as well as the Frontal_Inf_Orb_R and Cingulum_Ant_R/L, were found in the seizure recurrence group (SZR, Engel class II through IV). In LTLE, the difference between SZR and SZF outcome groups was much less marked. Parietal_Inf_L, Postcentral_L (hypometabolism, blue), and Cingulum_Ant_R, Precuneus_R (hypermetabolism, red) associated with an unfavorable outcome.

All patients exhibited hypometabolism in the temporal cortex ipsilateral to the epileptogenic region (Figure 1). Hypometabolism predominated in the temporal lobe in the SZF cohort, with some patients also exhibiting minimal hypometabolism or hypermetabolism outside the temporal lobe. By contrast, larger differences were observed in extratemporal areas in SZR cohort patients. Compared to healthy controls, volume of metabolic abnormalities measured in temporal areas, extratemporal areas and whole brain have differently correlated with surgical outcome, and relative to lateralization of TLE. In RTLE, relative to the SZF cohort, SZR cohort showed larger volume metabolic abnormalities in extratemporal areas (median, 11,060 vs. 2,112 mm^3^; *p* = 0.02). The metabolic abnormality volumes for temporal lobe and whole brain were larger in SZR (median, 3,356 and 14,252 mm^3^) than in SZF cases (median, 936 and 4,200 mm^3^) but the differences were not significant (*both p* > 0.05). In LTLE, the same trend was seen as in RTLE patients but no significant volume differences were found in temporal lobe, extratemporal areas and whole brain between SZR and SZF abnormal cerebral metabolism (temporal lobe: 1,056 vs. 1,040 mm^3^; extratemporal areas: 5,872 vs. 2,560 mm^3^; whole brain: 5,872 vs. 3,464 mm^3^; *all p* > 0.05). These results were in part consistent with focal or widespread hypometabolism classification by visual assessment on FDG-PET. No significant difference in volume of ipsilateral temporal hypometabolism was observed in both sides TLE (Mann-Witney test, *p* > 0.05) (Table 1). Those suggest that hypometabolism volume in TLE foci had no effect on surgical outcome, while the volume of extratemporal metabolic abnormalities could affect surgical outcome especially in RTLE. Larger volume of extratemporal metabolic change correlated with worse surgical prognosis. The critical volume of metabolic change outside RTLE foci (calculated from ROC curve) was 12,580 mm^3^.

### Intensity of Metabolic Abnormalities in Temporal and Extratemporal Areas

**Figure 2:**
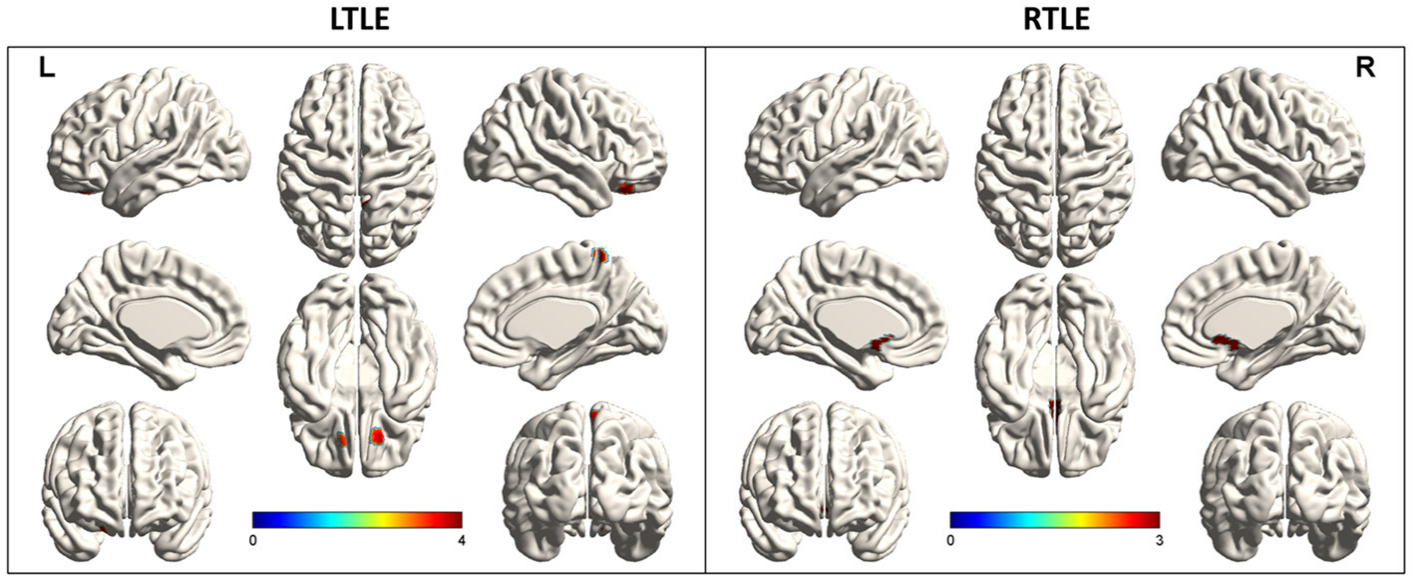
Metabolic differences according to outcome in right (R) and left (L) temporal lobe epilepsy (TLE) (*p* < 0.005). Areas of significant hypermetabolism in seizure recurrence (SZR) compared to seizure-free (SZF) differed between RTLE and LTLE. Differences in temporal lobe and extratemporal region were not strong enough to be visualized on SPM images in both RTLE and LTLE. Differences in temporal lobe area were insufficient for detection on SPM images in either RTLE or LTLE even when the threshold was lowered to *p* = 0.005. Hypermetabolism (red) in Precuneus_R, Frontal_Sup_Orb_R/L of LTLE and Cingulum_Ant_L/R of RTLE was evident when the threshold was set to *p* = 0.005.

Compared to healthy controls, hypometabolism was predominant in the ipsilateral temporal lobe (mesial, lateral and polemesial, lateral and pole). Peak intensity of hypometabolism in RTLE was −7.61 for SZR, −7.14 for SZF. Values of LTLE were −6.78 for SZR and −7.98 for SZF. When comparing SZR to SZF groups, differences in temporal lobe area were insufficient for detection on SPM images in either RTLE or LTLE even if the threshold was adjusted to *p* = 0.005 (Figure 2).

In addition to hypometabolism in temporal lobe foci, SZR cohort might show multiple hypo- or hyper-metabolism areas outside the temporal lobe. Compared to healthy controls, LTLE patients exhibited the most severe abnormalities in Parietal_Inf_L, Postcentral_L (hypometabolism, peak intensity −5.16, −4.58) and Cingulum_Ant_R, Precuneus_R (hypermetabolism, peak intensity 5.74, 4.62) in the SZR cohort. In RTLE patients, the greatest discrepancy was found in the Frontal_Inf_Orb_R (hypometabolism, peak intensity −3.67) and Cingulum_Ant_R/L (hypermetabolism, peak intensity 4.88, 4.17) in the SZR cohort (Figure 1).

TLE patients had hypometabolism in the temporal cortex ipsilateral to the epileptogenic region, but surgical outcomes were not associated with volume and intensity of ipsilateral temporal hypometabolism. SZR correlated with extratemporal metabolic profile that differed according to TLE lateralization.

### Special Extratemporal Brain Areas of Metabolic Abnormalities

**Figure 3:**
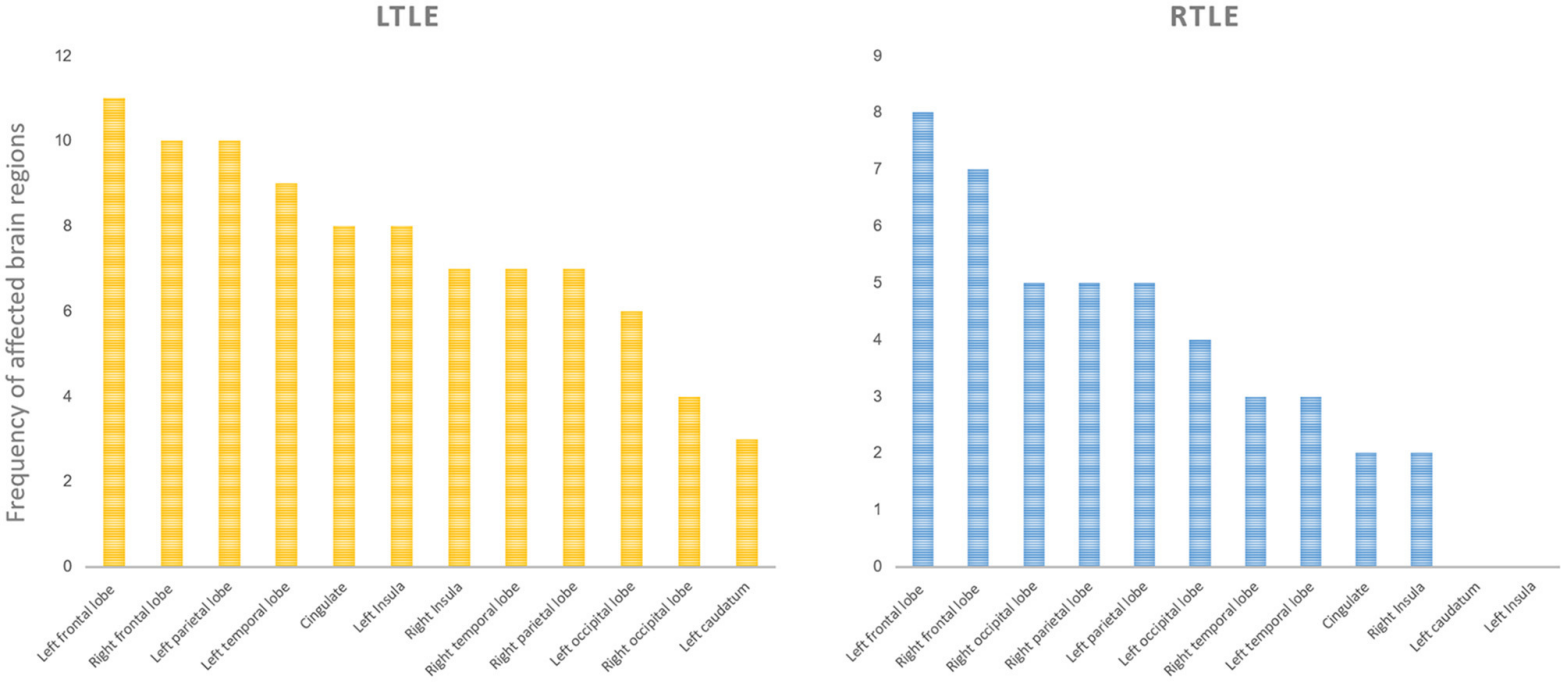
Involvement frequency of extratemporal region in all seizure recurrence (SZR) patients compared to the seizure-free (SZF) group. We compared the frequency of involvement of each extratemporal region of all patients in the SZR group. In both right (R) and left (L) temporal lobe epilepsy (TLE), the bilateral frontal lobe and left parietal lobe involvement frequencies were in the top three. Other areas included cingulate gyrus, bilateral occipital lobe, contralateral temporal lobe, insula, and caudate nucleus.

Comparing each SZR patient to the SZF cohort revealed the frequency of involvement of each extratemporal region of all SZR patients. In both LTLE and RTLE, the frequencies of bilateral frontal lobe and left parietal lobe involvement figured in the top three areas. Other areas included cingulate gyrus, bilateral occipital lobe, contralateral temporal lobe, insula, and caudate nucleus (Figure 3). However, comparing glucose uptake values between the two surgical outcome cohorts, we found that the peak intensity of metabolic changes in SZR patients differed with respect to comparisons with healthy controls. SZR vs. SZF differences were not strong enough to be visualized on SPM images at the fixed threshold. Lowering the threshold to *p* = 0.005 revealed LTLE hypermetabolism in Precuneus_R, Frontal_Sup_Orb_R/L, and RTLE hypermetabolism in Cingulum_Ant_L/R, but the difference in temporal lobe area was insufficient for detection (Figure 2).

## Discussion

Here we describe a large group of patients with a homogeneous clinicopathologic syndrome undergoing standard AMTR for TLE at a single center with a long postoperative follow-up. Metabolic abnormalities both in temporal and extratemporal areas were visualized and quantified using FDG-PET. We showed that the volume and intensity of metabolic changes in extratemporal areas and special brain areas assessed by FDG-PET correlated with a substantial proportion of surgical failures. We went further, to explore surgical outcomes in TLE with specific combinations of clinical characteristics. Hemispheric asymmetry was found to correlate with surgical results. Histopathology and MRI assessment of HS, as previously described (14, 25), may have little to do with the prognosis of surgery.

At the epilepsy center where this study was undertaken, an MRI normal hippocampus is an indication for FDG-PET evaluation even when other non-invasive evaluations lateralize to one temporal lobe foci. In this study, for some cases in which ictal onset localized to temporal lobe and an AMTR was performed, the neuronal loss in non-HS was milder than typically observed for mesial temporal sclerosis, but it does not indicate a higher rate of post-operation seizure control (26), on the contrary, sometimes negative MRI could prevent epilepsy surgeons from discovering temporal lobe epileptic foci. In TLE that is resistant to surgery, our glucose metabolism study has suggested an epileptogenic involvement of cortical areas outside temporal structures, both in cases with HS and non-HS. Accordingly, we showed that the volume and intensity of metabolic changes in temporal lobe foci had no effect on surgical outcomes. Previously, TLE patients with greater maximal temporal asymmetries were found to be less likely to achieve seizure-free status (27), but this analysis did not consider effects of epileptic foci and other brain regions on the contralateral temporal lobe, resulting in a reduction of the asymmetry index. Altogether, our results and other multivariate studies suggest that ipsilateral temporal hypometabolism may not be a prognostic factor, but rather a diagnostic indicator (13, 28).

The reasons for surgery failure are complex and variable, but emerging evidence points to failure to resect epileptogenic areas either within or outside the operated temporal lobe (7, 29–31). This situation can be defined as a dual pathology combining an extratemporal epileptogenic lesion and temporal epileptogenic zone (32, 33). Our results may be better explained by attributing epileptogenic potential to sites of metabolic abnormalities in extratemporal areas that were not included in resection. Several studies have reported extratemporal involvement associated with poor postoperative outcome, including nearby structures outside the standard resective margins or distant neocortical areas (13, 14, 25, 34). We found worse postoperative outcomes, especially among RTLE patients, correlated with larger volume or visual assessment range of metabolic changes in extratemporal areas detected by FDG-PET imaging.

Surgical failures in TLE have pointed to a subset of patients with primary seizure onset in the temporal lobe plus an “epileptogenic zone” that extends to nearby structures outside the standard resective margins, termed temporal-plus epilepsy (TPE) (5, 7, 34). Generally, extra-temporal targets are selected on the basis of alternative hypotheses formulated regarding the location(s) and extent of the epileptogenic zone(s) (35). The most frequently investigated brain regions are the temporo-parieto-occipital junction, fronto-basal and orbito-frontal cortex, suprasylvian operculum, and insula. Similarities in seizure recurrence rates as well as visual assessment of FDG-PET images between the extensive hypometabolism described in this study and TPE subgroups (7) suggest the same factors behind failed RTLE surgery. Except for nearby brain areas of foci, however, almost all other cortical areas could be targeted (34). In addition, for LTLE we found that while FDG-PET visual assessment and volume of metabolic change in extratemporal areas did not predict surgical outcomes, special cortical metabolic change in extratemporal areas could be predictive. Outcome predictions for LTLE patients were more challenging than for RTLE patients, related to ranges of predictive factors and this may account for the hemispheric asymmetry in metabolic patterns with respect to outcomes.

Such an explanation for surgical failure implies that a distributed epileptogenic network rather than a single epileptogenic focus may underlie surgically refractory epilepsy. Identification of epileptogenic foci and influences on networked extra-focal areas responsible for continued or recurrent postoperative seizures remain at the forefront of research to improve outcomes (36, 37). In addition to larger volume metabolic abnormalities in extratemporal areas linked to unfavorable outcomes, we describe special patterns associated with TLE surgical failure ranging from the frontal, parietal to occipital lobe and contralateral networks; some of which are consistent with electroclinical patterns corresponding to anterior and posterior spread (25). The present study's data support potential network theory of epileptogenicity in which nodes outside the seizure onset zone are implicated in seizure generation (37, 38). In TLE, the profile of metabolic abnormalities in extratemporal areas including volume, intensity and involvement of special brain areas assessed by FDG-PET, may indicate that these extratemporal limbic nodes are critical for epileptogenic network onset (39). Going forward, these should be sampled when an intracranial electroencephalography study of presumed temporal onset is performed. A shift toward treating the seizure network rather than a seizure-onset focus could alter the surgical and neuromodulatory management of focal epilepsy and guide electrode placement.

To address this issue therapeutically, extending resection to include nearby structures is one possible approach, when the metabolic pattern includes dispensable cortical areas such as the non-dominant lateral temporal cortex. When extended resection is not possible (e.g., dominant temporal lobe, frontal neocortex, or cerebral cortex motor area), treatment might involve placement of responsive neurostimulation electrodes at these extratemporal limbic nodes. Responsive neurostimulation electrodes would allow long-term recordings to validate nodes that are responsible for recurrent seizures as well as determine whether these sites are responsive to neuromodulation (40). When extratemporal areas such as the parietal motor area or occipital lobe (RTLE: Cingulum_Ant_R/L, Frontal_Inf_Orb_R; LTLE: Cingulum_Ant_L/R, Parietal_Inf_L, Postcentral_L, and Precuneus_R) are identified as metabolic change network nodes, it is essential to carefully consider possible adverse outcomes of resection, ablation or neurostimulation. While AMTR may likely fail for such patients, the effectiveness of possible alternative approaches is not yet clear. Advanced functional neuroimaging techniques have identified different phenotypes within the TLE spectrum, and specific metabolic patterns have poor surgical outcomes (41). Both extended resection and responsive neurostimulation treatment approaches will require FDG-PET neuroimaging for treatment planning and clear definition of projected network nodes.

This study had several limitations. First, this was a retrospective analysis and all patients underwent FDG-PET scan, leading to a low proportion of class I Engel outcomes (60%) especially among LTLE patients (43%). As patients without FDG-PET imaging were not included, which might have led to some selection and ascertainment biases. The decision to undergo surgery for epilepsy is complex, and involves consideration of the patient's baseline disease burden and overall clinical picture. It is not solely based on the probability of achieving freedom from seizures. Our findings are not meant to replace clinical judgment, but rather to assist decision making by providing an objective estimate of one key decision-driving factor-postoperative seizure-recurrence. Finally, preoperative video-EEG data, structural MRI neuroimaging and results of more sophisticated diagnostic tests such as ictal SPECT and invasive electroencephalogram were not analyzed in study patients who failed TLE surgery.

In conclusion, Extratemporal metabolic profiles detected by FDG-PET, in particular volume and intensity and affected special extratemporal brain areas may be associated with unfavorable postoperative seizure outcome in TLE and should therefore be considered in predictive models for epilepsy surgery. For TLE confirmed to have specific extratemporal metabolic abnormalities on FDG-PET, AMTR appears very unlikely to control seizures and should not be advised.

## Data Availability Statement

The data analyzed in this study is subject to the following licenses/restrictions: The dataset used and analyzed for the current study is available from the corresponding author on reasonable request. Requests to access these datasets should be directed to Shuo Hu, hushuo2018@163.com.

## Ethics Statement

The studies involving human participants were reviewed and approved by Ethical Commission of Medical Research Involving Human Subjects at Region of Xiangya Hospital, Central South University, China. Written informed consent to participate in this study was provided by the participants' legal guardian/next of kin. Written informed consent was obtained from the individual(s), and minor(s)' legal guardian/next of kin, for the publication of any potentially identifiable images or data included in this article.

## Author Contributions

YT and GL designed the method, acquisition of data, and prepared the manuscript. SH, LF, and BT designed the method, aided in data analysis, revised, and approved the manuscript. JL, TL, YL, and DC aided in data acquisition and interpretation. All authors contributed to the article and approved the submitted version.

## Conflict of Interest

The authors declare that the research was conducted in the absence of any commercial or financial relationships that could be construed as a potential conflict of interest.

## Abbreviations

AAL: automated anatomical labeling
AMTR: anteromedial temporal resection
EEG: electroencephalogram
FDG-PET: fluorine-18 fluorodeoxyglucose positron emission tomography
HS: hippocampal sclerosis
ILAE: International League Against Epilepsy
IQR: interquartile range
LTLE: left temporal lobe epilepsy
MNI: Montreal Neurological Institute
MRI: magnetic resonance imaging
ROC: receiver operating characteristic
RTLE: right temporal lobe epilepsy
SD: standard deviation
SPECT: single photon emission computed tomography
SPM 12: statistical parametric mapping
SZF: seizure free
SZR: seizure recurrence
TLE: temporal lobe epilepsy
TPE: temporal plus epilepsy.
